# The Multidimensional Efficiency of Pension System: Definition and Measurement in Cross-Country Studies

**DOI:** 10.1007/s11205-015-1017-3

**Published:** 2015-06-25

**Authors:** Filip Chybalski

**Affiliations:** Department of Management, Lodz University of Technology, Piotrkowska 266, 90-924 Lodz, Poland

**Keywords:** Pension, Retirement, Efficiency, Labour market, Cluster analysis

## Abstract

The existing literature on the efficiency of pension system, usually addresses the problem between the choice of different theoretical models, or concerns one or few empirical pension systems. In this paper quite different approach to the measurement of pension system efficiency is proposed. It is dedicated mainly to the cross-country studies of empirical pension systems, however it may be also employed to the analysis of a given pension system on the basis of time series. I identify four dimensions of pension system efficiency, referring to: GDP-distribution, adequacy of pension, influence on the labour market and administrative costs. Consequently, I propose four sets of static and one set of dynamic efficiency indicators. In the empirical part of the paper, I use Spearman’s rank correlation coefficient and cluster analysis to verify the proposed method on statistical data covering 28 European countries in years 2007–2011. I prove that the method works and enables some comparisons as well as clustering of analyzed pension systems. The study delivers also some interesting empirical findings. The main goal of pension systems seems to become poverty alleviation, since the efficiency of ensuring protection against poverty, as well as the efficiency of reducing poverty, is very resistant to the efficiency of GDP-distribution. The opposite situation characterizes the efficiency of consumption smoothing—this is generally sensitive to the efficiency of GDP-distribution, and its dynamics are sensitive to the dynamics of GDP-distribution efficiency. The results of the study indicate the Norwegian and the Icelandic pension systems to be the most efficient in the analyzed group.

## Introduction

Nowadays, when the demographics dividend is no longer in existence, adequacy of pension ceased to be the only criterion according to which pension systems are evaluated and compared in cross-country studies. The efficiency has gained no less importance. However, comparisons of pension systems functioning in different countries are more common in terms of adequacy, less in terms of efficiency. It may be caused by the shortage of synthetic measures evaluating the overall efficiency of a pension system. Why has studying the pension system efficiency become so important? Many countries facing demographic crisis, decide to reform their pension systems. And the crucial question is what should be the direction of the reform. Should the pension system become more funded or remain unfunded? Should they be more based on defined contribution or on defined benefit? More Beveridgian, or more Bismarckian? The analysis of efficiency supports the search of the answer to this question.

The literature review supplies some examples in which the efficiency of the pension system is (next to adequacy) the main criterion according to which opposite models of the pension system are compared and evaluated. The point is what combination of models mentioned above is the most effective one. This question is asked by many economists in their studies; however it is really difficult to find a precise definition of the efficiency of a pension system in a macro scale. Authors usually discuss many factors influencing the efficiency of pension system and the influence of pension system on the efficiency of the economy and labour market (e.g. Barr 1987; Hayek 1960; Boldrin and Montes 2009; Breyer and Kolmar 2002; Fenge and von Weizsäcker 2010; Le Garrec 2014). Such an approach is usually embedded in an overlapping generations model (OLG) and enables searching for the optimal parameters of a pension system, or simulating some changes of these parameters and their impact on the economy or labour market. Many researchers base their studies on theoretical pension systems. For instance, Weizsacker (2003) compares the Hayek, the Bismarckian and the Beveridgian pensions and searches for their best combination. Ayede (2010) addresses the problem of the increase in generational selfishness in parametric reforms of pay-as-you-go system as a potential result of the time inconsistency problem in optimal policies. In turn, Breyer and Kolmar (2002) analyze the effects of labour market integration on pay-as-you-go system provided that the labour is imperfectly mobile. Wrede (1998) searches for Pareto efficiency in a pay-as-you-go system with the use of a three-period OLG model. Hansen and Lonstrup (2009) analyze the optimal legal retirement age in an OLG model with endogenous labour supply. However, such analyses are usually limited to the evaluation of one or two given models of a pension system, very often theoretical, not empirical.

The contribution of this paper is to provide a solid and wide framework for the analysis of the multidimensional efficiency of empirical pension system in the way enabling cross-country comparisons. Such an approach to this problem is very rarely addressed in the existing literature, especially in terms of multidimensionality. The objective of the study arises from the need to search for better models (regimes) of pension systems in terms of two main criteria: adequacy and efficiency. My approach is based mainly on the proposition of some indicators, which would be useful in efficiency evaluation of different pension systems.

The main results of the study are as follows. Nine static, and three dynamic indicators of pension system efficiency are proposed. The static indicators are grouped in four separated sets, concerning: GDP-distribution efficiency, efficiency of ensuring pension adequacy, the efficiency at which pension system influences labour market, and cost efficiency. The dynamic indicators create a separated set and they concern the efficiency of adequacy, however in a dynamic view. This particular treatment of adequacy, static and dynamic, arises from the fact that adequacy is said to be the main result of a pension system functioning. The empirical verification, based mainly on Spearman’s rank correlation coefficient and cluster analysis, enables positive assessment of the proposed method.

The paper proceeds as follows. The next part includes literature review on the efficiency of pension system from the micro and macro perspective. Then, a new approach to defining and measuring pension system efficiency is presented. The third part of the paper includes the empirical verification of the proposed method. Here, statistical methods are employed: mainly Spearman’s rank correlation coefficient and cluster analysis. I use cross-sectional data containing 28 European countries in years 2007–2011. After the broad discussion of the verification results, the synthetic conclusions are drawn.

## The Efficiency of Pension System: Micro and Macro Perspective

The efficiency of pension system can be perceived, and thus defined, from several perspectives. The two most important ones result from two definitions of a pension system. The first one refers to the micro scale, in which pension system is a tool of income allocation or a tool of consumption smoothing in the life cycle (Barr 1987; Barr and Diamond 2006; Blake 2006; Góra 2008). The theoretical foundations for defining pension system in the micro scale are derived from the consumption and saving theories such as life cycle model (LCM) proposed by Modigliani and Brumberg (1954), or Ando and Modigliani (1963) and the permanent income hypothesis formulated by Friedman (1957). The common feature of both hypotheses is consumption smoothing by an individual, based on his or her expectations of the future development of certain economic parameters, including income. It should be noted however, that LCM indeed usually lays the foundations for the analyses of pension system and pension decisions from the perspective of an individual or a household. From the micro perspective, the optimal pension system ensures the maximization of the consumer utility across the life course.

The other perspective refers to the macro scale, in which a pension system is a tool for dividing the current GDP between the working generation and the generation of pensioners (Góra 2008). Such approach is also presented by Barr and Diamond (2006). In their opinion, future GDP is crucial in a pension system, since the consumption of future pensioners will be determined by what will be produced in the future, mainly by the generation of their children. This way, in the future, the generation of parents (already pensioners) and the generation of their children (already in the production age) will share the GDP between themselves. The pension system, as a tool of income distribution between generations, determines its rules, in a more or less fair and efficient manner, since this distribution has a major impact on economic development and, at the same time on the future GDP to be divided between the respective generations. Nevertheless, the rules of dividing GDP between generations should be correlated with the contribution made by a given generation to the pension system in the period of its economic activity. This is the reason why the overlapping generations models are broadly employed in pension system analyses.

According to the above mentioned definitions of a pension system, two perspectives for evaluating the efficiency occur—a micro- and macroeconomic one. From the micro perspective, the optimal pension system ensures the maximization of the consumer utility across the life course. The subject evaluating the efficiency is an agent. From his or her point of view, an efficient pension system is one which delivers the maximum benefit by a given contribution, or a given benefit by a minimized contribution, irrespectively whether the contribution is included in the tax, or it is separated from the tax (pension contribution). However, there is one important problem related to the perception of a real made contribution to the pension system during the working life. For a pensioner, it is much easier to evaluate his or her current pension in payment, while disregarding the contribution, or overestimating it.

Since the efficiency of a pension system from the perspective of an agent is the ratio between the amount of benefits and the amount of contributions, an appropriate measure of this efficiency seems to be the rate of return. However, direct comparison of returns between PAYG and fully funded scheme is misleading. In the case of PAYG, there are in fact two different rates of return. The first one is the rate at which contributions are indexed and only this rate is actually perceived by individuals. The other rate is the real rate of return, which according to actuarial conditions should be equal to the internal rate of return (IRR) since it ensures the balance between assets and liabilities of the scheme. If pension liabilities are indexed at this rate of return, the scheme remains balanced (Settergren 2002). As PAYG schemes (including NDC) are based on the government promise—politicians set the rules of indexation (see Barr and Diamond 2006)—the rate of indexation may be interpreted as the measure of government promise dynamics. The difference between the rate of indexation and the internal rate of return may be perceived as the measure of risk in PAYG since it expresses the difference between the promise and the real capacity of the scheme; the greater the difference, the higher the risk. This problem does not exist in the case of fully funded scheme where the only rate of return is the portfolio’s rate of return. This rate explains current changes in the valuation of portfolio assets. However, from the pensioner’s point of view, in fact, only the rate of return for the whole period of saving is important, i.e. the rate calculated at the moment of retirement, when the accumulated capital is transferred into life time annuity.

Based on the mentioned above definition of the efficiency of pension system from an agent’s point of view, this type of efficiency refers to the adequacy of pension system while the higher this efficiency is, the higher the adequacy of pension.

A quite different approach is desirable when analyzing the pension system efficiency from the macro perspective. It results from the fact that the way a given agent smoothes his or her consumption may affect the consumption smoothing of another agent and this relationship may influence GDP distribution. From the macro perspective the efficient pension system ensures the optimal GDP distribution between generations—today, but also in the future—through minimizing the negative impact the system has on the economy and labour market. It should be also emphasized that, in contrast to the micro scale, the efficiency in macro scale does not refers to the relation between accumulated (in the agent’s life cycle) benefits and contributions. From the macro perspective, the relation between aggregated flows between generations is crucial. Therefore, from this point of view, the efficiency is the relation between the degree to which the benefits for pensioners are ensured, and the cost of it, incurred by the working generation. In this paper, macro perspective is the leading one.

## A New Approach to the Measurement of Pension System Efficiency

### Definition

In this section, I make an attempt to answer the crucial question: how to define the efficiency of a pension system to enable objective comparisons in cross-section studies based on empirical (not model or theoretical) pension systems? The efficiency of empirical pension systems may be evaluated in two main contexts: internal and overall. The internal efficiency of pension system refers only to this system or its selected parts (tires, pillars), e.g. the efficiency of pension funds, the efficiency of PAYG scheme. It comprises only the administrative cost of pensions disregarding undesirable costs or effects (e.g. on labour market). Such efficiency is usually measured with the use of appropriate rates of return and the ratio of administrative costs. The overall efficiency of a pension system includes the abovementioned internal efficiency as well as external efficiency, characterizing the impact a pension system has on the economy, public finance and labour market. This impact may be perceived as the indirect, often undesirable, cost of a functioning pension system. However, as a feedback, it may deteriorate a pension system as well. For instance, when a pension system is expensive, too adequate, with a low effective retirement age, and needs high contributions, its impact on the labour market is negative, which may result in the decrease in incomes from contributions or taxes in the short as well as in the long run.

The above discussion leads to the conclusion that the overall efficiency is substantive when comparing pension systems since it is of a synthetic nature, taking into account the many aspects of the functioning of a pension system. Therefore, the efficiency of a pension system is perceived as the overall one (not only internal) and, by given conditions (mainly demographics), refers to the relation between pension system adequacy (including protecting against poverty and consumption smoothing), and its cost in the sense of inputs (including pension expenditure, administrative costs) as well as in the sense of side effects on the economy—especially on the labour market.

This definition refers mainly to the macro scale. It takes also into account the possible openness of a pension system, which has become more and more common in times of globalization. The openness of pension systems means the possibility of the income allocation in life course with the use of foreign economies or financial markets. Therefore, the assumption about the closed economy is unnecessary and Pareto-improving is possible since one generation may gain without harming the other one, but through opportunities arising from differences in economies growths or returns on financial markets in different countries. An opened pension system in a both static and dynamic approach, needs not to be a zero-sum game and in this game two generations in a given country may gain at the expense of a generation (or generations) in another country (or other countries).

### Idea of Measurement

My approach is based on empirical pension systems and statistical data describing their given categories referring to the efficiency. The evaluation does not need any unrealistic assumptions and may be based on a static or a dynamic approach. In the first case, cross-sectional data and in the other case, time series cross-sectional data are employed. The main advantage of presented approach is that it enables cross-country quantitative comparisons which serve for searching better pension system designs. The crucial thing is the selection of the variables describing conditions, inputs, outputs, and side effects of pension system, which are taken into account with reference to the overall pension system efficiency. However, if the evaluation of the efficiency is to be of cross-country character, the data used should meet the requirements of the international comparativeness. On the other hand, some data availability limitations also exist. Taking the above into account, the fallowing variables referring to the conditions, inputs, outputs and side effects of pension system, may be proposed:

- Conditions: old-age dependency ratio (ODR),
- Inputs: total current pension expenditure as the percentage of GDP (PE/GDP), administrative cost of public pension system (AC),
- Outputs: at-risk-of-poverty rate of pensioners (ARP), median relative income ratio of elderly people aged 65+ (MRI65+), aggregated replacement ratio (ARR),
- Side-effects: employment rate of people aged 55–64 (EMP55-64), employment rate of people aged 65–74 (EMP65-74),^1^ average effective age of retirement (ARA—average retirement age).

ODR indicator represents the main condition of pension system which has been the most important motivation for contemporary pension reforms. However, demographics is not actually the problem of pension systems and its financial sustainability. The fact that people live and stay healthy longer is a positive phenomenon. The problem is that people are not willing to accept the economic consequences of their longer and healthier life—leaving the labour market later. Demographics (represented by ODR) is supposed to be the only exogenous (or at least, the most exogenous) variable taken into account in the presented approach as it may affect the pension system and a pension system should not affect ODR. The other variables are endogenous since their changes may be affected by ODR or other variables considered below.

The inputs are represented by two indicators. The first one is PE/GDP which measures the proportion of GDP earmarked for the pensioner’s generation and, therefore, the macroeconomic cost of pensions. However the comparability features of this indicator in cross-country studies are limited as it does not take into account the demographic conditions (Chybalski 2014; Marcinkiewicz and Chybalski 2014). The other indicator is AC which measures the administrative costs in a public pension system. Both input indicators are destimulating efficiency factors—their lower value means lower inputs and thus greater efficiency.

The outputs are represented by four indicators measuring the pension adequacy. ARP is a destimulating variable (the-lower-the-better) and measures the ratio of pensioners’ population living under the poverty threshold. ARP may be calculated by different cut-off points: 40, 50, 60 or 70 % of median equivalised income, which enables the analysis of the dispersion around the at-risk-of-poverty threshold. The lower the ratio, the lower the poverty among the elderly. MRI65+ and ARR are the measures of the degree of consumption smoothing and they have the character of stimulants (the-grater-the-better). However, there is a significant difference between these two indicators. The first one provides for total income after tax divided by the number of the members of a household. The latter one takes into account gross incomes (gross pensions as the ratio of gross earnings). All the outputs indicators explain the main microeconomic objectives of pension system—poverty alleviation and consumption smoothing.

The other indicators, i.e. EMP55-64, EMP65-74 and ARA, measure the side effects of a pension system on the labour market. Obviously, the pension system may affect also the situation of younger cohorts in the labour market. However, this is quite a different problem concerning the competition between young and old on the labour market, discussed in many other works (e.g. Jousten et al. 2008; Kalwij et al. 2010; Boldrin et al. 1999; Hammermesh and Grant 1979; Hammermesh 1987). This issue is beyond the scope of this paper. Therefore, I focus on the age groups, whose decisions about retirement may be directly affected by the pension system. The proposed three side effects indicators are stimulants since their greater value means that pension system encourages people to leave labour market to a lesser extend or later.

All the indicators mentioned above comes from Eurostat database and are selected from the set of indicators monitoring the realization of the objectives of the Open Method of Coordination^2^ (excluding average effective age of retirement, which comes from OECD database). These indicators seem to be the most important when measuring the inputs and the outputs of pension systems as well as conditions and side effects of pension system functioning. The other indicators often include very similar information, or consider another age group (e.g. 60 and over, instead of 65 and over, however the latter is more consistent with the legal retirement age nowadays). Some indicators which are not included in the inputs or outputs sets refer to the income asymmetry or dispersion among pensioners which is not in fact the measure of pension adequacy in sense of consumption smoothing or poverty alleviation, and may result from the income disproportion in working period. Additionally, such indicators do not measure the efficiency in the sense of relation between inputs and outputs. They rather present the effect of intragenerational redistribution serving not only for poverty alleviation but also for smoothing incomes across a pensioner’s generation. On the basis of the presented indicators characterizing conditions, inputs, outputs and side-effects, two complementary approaches to the measurement and evaluation of pension system efficiency in cross-country studies are proposed: a static and a dynamic one.

### Efficiency Indicators—Static Approach

The abovementioned measures are the starting point for creating corresponding efficiency indicators based on the rule, that efficiency is mainly the ratio between outputs and inputs, however it also takes into account the side effects. Therefore, the following sets of indictors may serve for static evaluation of four dimensions representing overall efficiency of a pension system in cross-country studies.

#### *Dimension 1*

GDP-distribution efficiency includes one indicator (Chybalski 2014; Marcinkiewicz and Chybalski 2014):

$$GDP - D\_e = \frac{PE/GDP}{ODR}$$

where GDP_D_e denotes GDP-distribution efficiency indicator.

This indicator refers directly to the macroeconomic definition of a pension system and conditions, in which the distribution of GDP is realized. The old-age dependency ratio affects the GDP distribution. However, an efficient pension system should be resistant to the demographics as much as possible. This indicator measures this resistance since it is the ratio between pension expenditure as the percentage of GDP, and old-age dependency ratio. The lower the value of this indicator, the greater the resistance of pension system to demographic changes (i.e. to the aging of population).

#### *Dimension 2*

Adequacy efficiency measures the efficiency at which the adequacy is ensured in a pension system. This set includes three efficiency indicators:

$$ARP\_e = \frac{1/ARP}{PE/GDP}$$

$$MRI65 + \_e = \frac{MRI65 + }{PE/GDP}$$

$$ARR\_e = \frac{ARR}{PE/GDP}$$

where ARP_e, efficiency of poverty alleviation; MRI65+_e, efficiency of consumption smoothing measured by relative median income ratio; ARR_e, efficiency of consumption smoothing measured by aggregated replacement ratio.

 This set of indicators represents the main output of pension system functioning—ensuring incomes for pensioners. Thus, it refers to the adequacy of pensions. All the indicators included in set 2 are stimulants and, therefore, ARP_e includes the inverse of ARP indicator in the nominator. This means that their greater values correspond with greater adequacy efficiency of pension system in the sense of ensuring this adequacy.

#### *Dimension 3*

Labour market efficiency measures the most important side effect of pension system which is its impact on the labour market. What is very important, this impact is bidirectional since if a pension system affects labour market negatively, a labour market influences the pension system through lower incomes from pension contributions paid by workers. This set includes three indicators:

$$EMP55 - 64\_e = \frac{EMP55 - 64}{PE/GDP}$$

$$EMP65 - 74\_e = \frac{EMP65 - 74}{PE/GDP}$$

$$ARA\_e = \frac{ARA}{PE/GDP}$$

where EMP55-64_e, efficiency in terms of labour market I; EMP65-74_e, efficiency in terms of labour market II; ARA_e, efficiency in terms of labour market III.

All the indicators classified to this set, refer to two age-groups—the first one is directly before retirement, and the other one is directly after retirement. They are stimulants and measure how the GDP distribution affects the labour market. The greater the value of these indicators, the more positive (or less negative) the impact of pension system on the labour market. They all inform about the relation between the participation of elderly (directly before legal retirement age and after that) in the labour market (i.e. their participation in the production of GDP), and the share of GDP distributed to them through a pension system.

#### *Dimension 4*

Cost efficiency, including two indicators: AC1_e—administrative cost of pension system expressed as a percentage of pension benefits, AC2_e—administrative cost of pension systems expressed as a percentage of GDP:

$$AC1\_e = \frac{AC}{PE}$$

$$AC2\_e = \frac{AC}{GDP}$$

These indicators represent the inputs in a pension system and measure the ratio between administrative cost of ensuring benefits from public system, and expenditure on these benefits or GDP. Both indicators are destimulating factors of efficiency.

### Efficiency Indicators—Dynamic Approach

In accordance with the presented idea of the measurement of pension system efficiency, the output of its functioning is the broadly understood adequacy, containing poverty alleviation and consumption smoothing. The former is measured be ARP_e indicator, the latter by two indicators: RMI65+_e and ARR_e. Next to the static evaluation of adequacy efficiency (second dimension), no less important is also the dynamic one. Therefore, following three dynamic efficiency indicators of pension system, referring to its adequacy, may be proposed:

$$ARP\_de = \frac{{ARP_{0} - ARP_{t} }}{{\frac{{PE_{t} }}{{GDP_{t} }}:\frac{{PE_{0} }}{{GDP_{0} }}}}$$

$$RMI\_de = \frac{{RMI_{t} - RMI_{0} }}{{\frac{{PE_{t} }}{{GDP_{t} }}:\frac{{PE_{0} }}{{GDP_{0} }}}}$$

$$ARR\_de = \frac{{ARR_{t} - ARR_{0} }}{{\frac{{PE_{t} }}{{GDP_{t} }}:\frac{{PE_{0} }}{{GDP_{0} }}}}$$

where ARP_de, dynamic efficiency of poverty alleviation; RMI_de, dynamic efficiency of consumption smoothing measured by relative median income ratio; ARR_de, dynamic efficiency of consumption smoothing measured by aggregated replacement ratio.

In all the indicators two periods of time (0 and *t*) are compared, i.e. the difference between them. The indicators are constructed as to be the stimulants. Therefore, in the case of ARP_de, the nominator is conversed in comparison to other two indicators. In case of denominator, the ratio between pension expenditures as percentage of GDP is used instead of the difference in order to avoid the denominator equal to 0. The interpretation of the proposed dynamic efficiency indicators is as follows. ARP_de measures in fact the efficiency of poverty reduction. RMI_de and ARR_de measure the efficiency of the increase in the level of consumption smoothing. Their value >0 means the increase in the efficiency of poverty reduction or consumption smoothing, which may be accompanied by the lower, higher, or the same level of pension expenditures. However, as we especially consider the efficiency measurement in cross-country studies, for two countries with the same dynamics of pension expenditure, the greater efficiency has the country with grater poverty reduction or greater increase in the level of consumption smoothing. The indicators lower than 0 mean the inefficiency in the changes in poverty or in consumption smoothing.

## Empirical Verification of the Proposed Approach

### The Purpose

The approach to the measurement of pension system efficiency is based on four different dimensions, theoretically. However it is likely that some of these indicators classified to different sets, or even the whole dimensions, are similar when analyzing statistical data. Therefore, the proposed method of evaluation of pension system efficiency requires verification based on the empirical data. The main question to be answered is whether there are actually four different dimensions of pension system efficiency when using proposed indicators in its evaluation in cross-country studies. The other one is whether there is a significant difference between a static and dynamic approach to the measurement of the adequacy efficiency.

### Data and Methods

The study is based mainly on the Spearman’s rank correlation coefficient and cluster analysis (tree clustering). The Spearman’s rank correlation coefficient is employed for static as well as for dynamic indicators. It is used to identify the relationships between proposed efficiency indicators. I employ Spearman’s rank correlation coefficient since it is of nonparametric nature and measures the strength of association between two ranked variables. Its main advantage in comparison to Pearson’s correlation coefficient is the fact that it provides not only for linear, but also for others—nonlinear relationships.

Cluster analysis is run solely for static indicators, however not only for objects (as usually), but also for variables. From my point of view, the latter is no less important than the former since in order to answer the asked question concerning the number of efficiency dimensions, the proposed indicators require classification. If they create four clusters of variables, the proposed approach of measurement finds full justification. If the number of clusters is lower or greater than four, the method may need some modification in terms of defining dimensions or classifying indicators to these dimensions. However, the cluster analysis for objects, i.e. countries, is also conducted since it may deliver complementary information on the method. Namely, it enables testing the stability of the results in the time and identifying groups of similar pensions systems in terms of efficiency. Cluster analysis is preceded by the standardization of the data. The difference between objects and clusters is measured with the use of Euclidean distance. To link the objects into clusters, Ward’s method—based on the analysis of variance—is employed (Ward 1963).^3^

The analysis covers 28 European countries: Austria, Belgium, Bulgaria, Cyprus, Czech Rep., Denmark, Estonia, Finland, France, Germany, Greece, Hungary, Iceland, Italy, Latvia, Lithuania, Malta, Netherlands, Norway, Poland, Portugal, Romania, Slovakia, Slovenia, Spain, Sweden, Switzerland and the United Kingdom. I use cross-sectional data from the years 2007–2011. The sources of the data are Eurostat and OECD databases. From the latter comes only data on average effective age of retirement.^4^ The analyzed period is determined by the availability of data. For some countries the selected indicators are not available for 2012 at the moment when the study is conducted. The Spearman’s rank correlation coefficients are calculated for the whole analyzed period of time, and the cluster analysis is conducted for each year separately.

### Results

Table 1 presents the Spearman’s rank correlation coefficients between proposed efficiency indicators.

**Table 1:** Spearman’s rank correlation coefficient between efficiency indicators

	GDP-D_e	ARP_e	RMI65+_e	ARR_e	EMP55-64_e	EMP65-74_e	ARAm_e	ARAf_e	AC1_e	AC2_e
GDP-D_e	1.00	−0.16	−**0.67**	−**0.62**	−**0.69**	−**0.36**	−**0.82**	−**0.84**	**0.34**	**0.50**
ARP_e	−0.16	1.00	**0.63**	**0.65**	0.04	−**0.18**	**0.26**	**0.26**	0.03	−0.01
RMI65+_e	−**0.67**	**0.63**	1.00	**0.88**	**0.49**	**0.25**	**0.85**	**0.85**	−**0.32**	−**0.51**
ARR_e	−**0.62**	**0.65**	**0.88**	1.00	**0.48**	**0.22**	**0.77**	**0.77**	−**0.42**	−**0.53**
EMP55-64_e	−**0.69**	0.04	**0.49**	**0.48**	1.00	**0.69**	**0.77**	**0.77**	−**0.21**	−**0.30**
EMP65-74_e	−**0.36**	−**0.18**	**0.25**	**0.22**	**0.69**	1.00	**0.50**	**0.47**	−**0.45**	−**0.45**
ARAm_e	−**0.82**	**0.26**	**0.85**	**0.77**	**0.77**	**0.50**	1.00	**1.00**	−**0.42**	−**0.63**
ARAf_e	−**0.84**	**0.26**	**0.85**	**0.77**	**0.77**	**0.47**	**1.00**	1.00	−**0.42**	−**0.62**
AC1_e	**0.34**	0.03	−**0.32**	−**0.42**	−**0.21**	−**0.45**	−**0.42**	−**0.42**	1.00	**0.90**
AC2_e	**0.50**	−0.01	−**0.51**	−**0.53**	−**0.30**	−**0.45**	−**0.63**	−**0.62**	**0.90**	1.00

Correlation coefficients statistically significant at the significance level 0.05 are bolded

The GDP-D_e indicator (destimulating factor) is generally correlated with other indicators as it could be expected. In the case of employment efficiency indicators, the Spearman’s correlation coefficient is negative which means that the higher the GDP-distribution efficiency (the lower value of that indicator), the higher the labour market efficiency. Since the GDP-D_e indicator measures the resistance of pension system to demographic changes, this resistance influences the labour market efficiency positively. In the case of cost efficiency, the positive correlation is reported since both AC1_e and AC2_e indicators, as well as GDP-D_e indicator, are destimulating factors—the lower their value, the higher the efficiency. However, in the case of one pair of variables—GDP-D_e and ARP_e, the Spearman’s correlation coefficient is to some extent surprising. Its value is very low and statistically insignificant which means the lack of the relationship between these two indicators. This suggests that the efficiency of ensuring poverty alleviation in pension system is resistant to the changes in the relation between pension expenditures and demographics. I think it is a positive phenomenon since the main goal of pension systems is to ensure the minimal required level of consumption after retirement. However, as the efficiency of ensuring consumption smoothing is concerned, which is measured by of RMI65+_e and ARR_e, the GDP-distribution efficiency affects it positively—the higher the GDP-distribution efficiency (the lower value of its indicator), the more effective the consumption smoothing measured by the ratio between aggregated replacement rate or relative median income ratio, and pension expenditures (as % of GDP). Since the adequacy efficiency indicators and labour market efficiency indicators are constructed as stimulants, they are in the most positively correlated. However, the correlation between ARP_e indicator and EMP55-64_e is statistically insignificant (almost equal to 0), and between ARP_e and EMP65-74 negative (but very low). This means some independence between poverty alleviation and consumption smoothing, although these both phenomena reflects the same dimension of pension system efficiency—efficiency of ensuring the adequacy. GDP-D_e and AC_e indicators, as destimulating factors, are negatively correlated with all the stimulating factors.

**Fig. 1:**
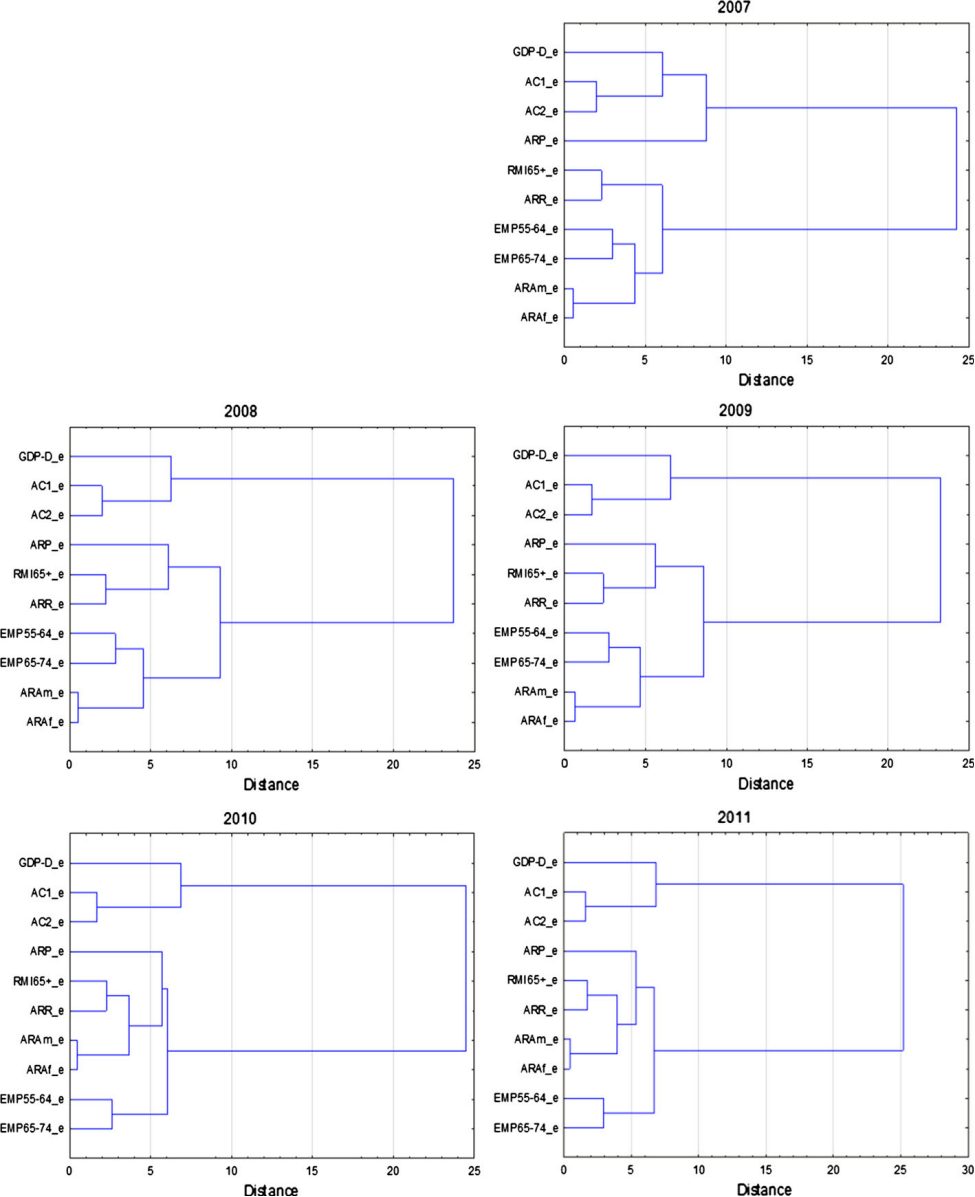
Cluster analysis for the efficiency indicators

The cluster analysis enables delivering other results concerning proposed sets of efficiency indicators (see Fig. 1). It confirms that pension system efficiency is of multidimensional nature. Generally, 4–5 separated clusters are observed. The first one includes one variable—GDP-D_e, representing the dimension referring to the resistance of a pension system to demographic factors. The second cluster consist of two indicators (AC1_e and AC2_e), referring to cost efficiency. The third cluster includes labour market efficiency indicators, however in 2010 and 2011 some changes in this group are observed. Namely, the indicators reflecting the average age of retirement (ARA_m and ARA_f) become more similar to indicators representing consumption smoothing efficiency (RMI65+_e and ARR_e). It suggests that the level of consumption smoothing becomes more and more linked to the decision on when to retire. In all the analyzed years, a separate group is created by ARP_e which confirms that the adequacy of pension system has at least two sub-dimensions—poverty alleviation and consumption smoothing. The similarity of two indicators concerning employment rates (EMP55-64_e and EMP65-74_e) is still very high and stable, and these indicators may be treated as a separated cluster, especially in 2010 and 2011.

The dynamics of cluster structure in the whole analyzed period is surprising to some extent however, when providing greater distance to identify clusters, two main groups of indicators arises. The first one reflects the first and the fourth dimension of pension system efficiency, and the other one the second and third dimension. By the lower level of cut-off-point of distance, four or five sets of indicators appear, to a large extent consistent with the proposed four dimensions of pension system efficiency. It is worth emphasizing that the distance for the last agglomeration (determining the cluster containing all the indicators) remains generally stable over the whole analyzed period and equals about 25.

**Fig. 2:**
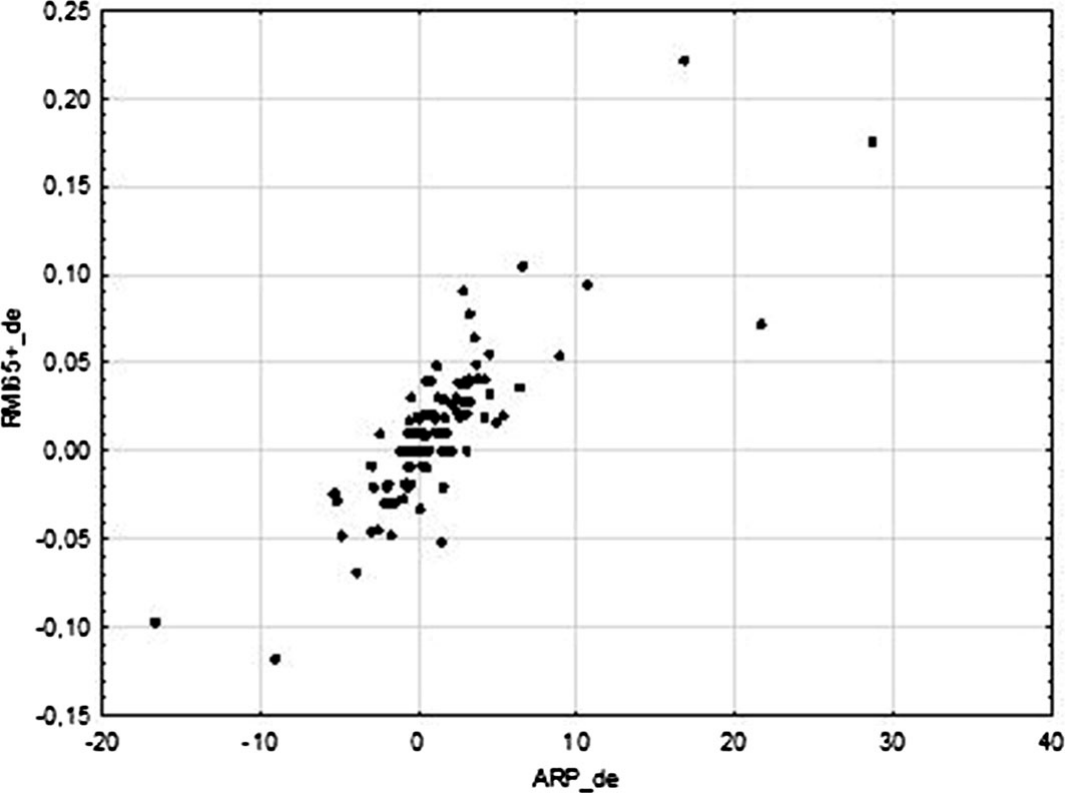
Correlation plot for the two dynamics efficiency indicators: ARP_de and RMI65+_de

The results presented in Table 2 confirm the justification for applying two approaches to the efficiency evaluation: a static and a dynamic one. Spearman’s correlation coefficients are statistically significant for two separated groups of indicators: the first one—measuring static efficiency, and the other one—measuring dynamic efficiency. The correlations between these two groups are statistically insignificant. This means that there is no relationship between the static and dynamic measurement. More precisely, the efficiency of reduction poverty is not correlated with the efficiency of protecting against poverty, and the efficiency of the increase in the level of consumption smoothing is not correlated with the level of consumption smoothing. In the group of dynamic indicators, two correlation coefficients are worth emphasizing: between ARP_de and RMI65+_de, as well as between ARP_de and ARR_de. The first one suggests that the increase in the efficiency of poverty reduction highly corresponds with the increase in the efficiency of consumption smoothing measured by RMI_65+. ARR indicator is also significantly and positively correlated with ARP_de, however incomparably weaker, although refers to the dynamic efficiency of consumption smoothing as well. Figure 2 confirms high correlation between the efficiency of poverty reduction and the increase in the efficiency of consumption smoothing measured by RMI65+, and suggests that this relationship is linear.

**Table 2:** Spearman’s rank correlation coefficient between selected efficiency and dynamic efficiency indicators (calculated for the years 2008–2011)

	ARP_e	RMI65+_e	ARR_e	ARP_de	RMI65+_de	ARR_de
ARP_e	1.00	**0.65**	**0.66**	0.08	0.13	0.01
RMI65+_e	**0.65**	1.00	**0.87**	0.12	0.12	0.07
ARR_e	**0.66**	**0.87**	1.00	0.11	0.16	0.11
ARP_de	0.08	0.12	0.11	1.00	**0.81**	**0.46**
RMI65+_de	0.13	0.12	0.16	**0.81**	1.00	**0.49**
ARR_de	0.01	0.07	0.11	**0.46**	**0.49**	1.00

Correlation coefficients statistically significant at the significance level 0.05 are bolded

The differences between Spearman’s rank correlation coefficients for given pair of indicators in Tables 1 and 2 results from the fact that in the latter case, static indicators cover the shorter period of time (2008–2011). This results from calculating dynamic indicators (their number for a given object is reduced by 1)

**Fig. 3:**
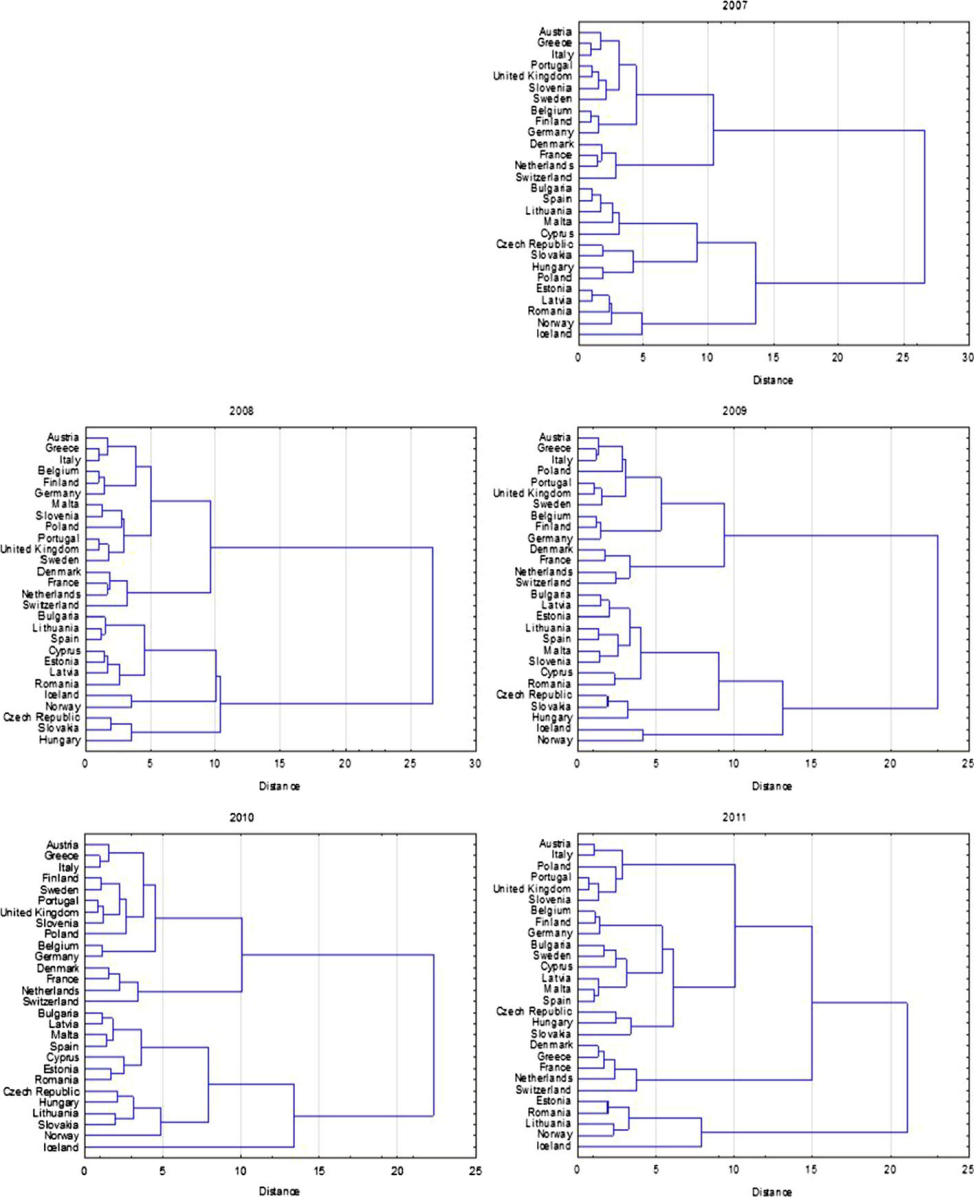
Cluster analysis for countries

The next empirical verification for the proposed approach to the measurement of pension system efficiency is based on the second cluster analysis, however this time not for variables, but for the objects—country pension systems. The proposed static efficiency indicators here are the variables differentiating pension systems in terms of efficiency (see Fig. 3). The number of clusters containing different countries, pension systems, seems to be stable over time and oscillate between 5 and 6. The Following groups of countries may be identified:

- Group 1: Austria, Greece (until 2010), Italy, Portugal, UK, Slovenia (excluding 2009), Sweden (until 2010), Poland (excluding 2007), Belgium (until 2010), Finland (until 2010), Germany (until 2010);
- Group 2: Denmark, France, Netherlands, Switzerland, Greece (2011);
- Group 3: Bulgaria, Spain, Lithuania, Malta (excluding 2008), Cyprus;
- Group 4: Estonia, Latvia, Romania, Norway;
- Group 5: Czech Rep., Hungary, Slovakia, Poland (2007);
- Group 6: Iceland.

Some countries are very close to each other in terms of efficiency over the whole analyzed period of time, closer than other countries in selected groups. There are:

- Denmark, France, Netherlands and Switzerland;
- Austria and Italy;
- Belgium, Finland and Germany;
- Czech Rep., Slovakia and Hungary.

When aggregating static efficiency indicators, Norway, Iceland, Slovakia, Czech Rep., Romania, Switzerland and Lithuania, should be classified to the group of the best pension systems among analyzed. There are mainly countries from groups 4–6 in cluster analysis. The worst pension systems among analyzed in terms of the static efficiency function in: Austria, France, Greece, Italy, Malta, Portugal, UK and Slovenia. These are mainly countries classified to the group 1. The in-depth analysis of the most and the least efficient pension systems is not the objective of this paper. However, it is worth emphasizing briefly that Norway and Iceland have definitely the most efficient pension systems among analyzed countries. They are classified as the best ones every year. What is the main strength of them? Although they are relatively expensive (especially Icelandic one), they have very high indicators referring to the efficiency of consumption smoothing and labour market. The worst pension system is definitely the Italian one. It is not very expensive indeed, however very inefficient in terms of consumption smoothing and labour market. This system has also low efficiency of poverty alleviation.

The analysis of three dynamic efficiency indicators enables indicating pension systems which improve their adequacy efficiency and deteriorate it mostly. As far as poverty alleviation is concerned, the fastest increase in efficiency is reported by Baltic states: Lithuania, Latvia and Estonia. And the fastest decrease in this type of efficiency is observed in Sweden, Poland and Bulgaria. In terms of consumption smoothing, the fastest increase in efficiency characterizes Lithuania, Romania and Latvia, and the fastest decrease is observed in Sweden, Poland and Switzerland. When comparing these groups with the groups of the best and worst pension systems in static view, they differ significantly. This confirms that the static and dynamic approach to the measurement, give quite different results, therefore they should be treated as complementary ones, not alternative.

The last but not least point of empirical verification of the proposed approach to the measurement of pension system efficiency involves the Spearman’s rank correlation analysis of the dynamics of static efficiency indicators, obviously completed by dynamic efficiency indicators. In order to do this, for all the static indicators, indices of dynamics for the whole analyzed period are calculated (as the ratio between the value of a given indicator in 2011 and in 2007), as well as three dynamic indicators, referring to the adequacy (also for the whole period). The results presented in Table 3 enable the assessment of possible relationships not between the levels of indicators, but of the changes in their levels.

**Table 3:** Spearman’s rank correlation coefficient between indices of static efficiency indicators and dynamic efficiency indicators

	GDP-D_e	ARP_e	RMI65+_e	ARR_e	EMP55-64_e	EMP65-74_e	ARAm_e	ARAf_e	AC1_e	AC2_e	ARP_d	RMI_d	ARR_d
GDP-D_e	1.00	0.11	−**0.55**	−0.21	−**0.82**	−**0.77**	−**0.93**	−**0.91**	−0.32	0.03	**0.42**	**0.40**	**0.51**
ARP_e	0.11	1.00	**0.60**	**0.58**	−0.18	−0.13	−0.17	−0.16	−0.31	0.26	**0.81**	**0.84**	**0.70**
RMI65+_e	−**0.55**	**0.60**	1.00	**0.77**	**0.46**	**0.46**	**0.51**	**0.51**	−0.19	−0.06	0.31	**0.45**	0.25
ARR_e	−0.21	**0.58**	**0.77**	1.00	0.16	0.09	0.19	0.20	−0.29	−0.04	**0.45**	**0.49**	**0.61**
EMP55-64_e	−**0.82**	−0.18	**0.46**	0.16	1.00	**0.85**	**0.89**	**0.90**	0.32	−0.11	−**0.49**	−**0.45**	−**0.54**
EMP65-74_e	−**0.77**	−0.13	**0.46**	0.09	**0.85**	1.00	**0.81**	**0.84**	0.33	−0.12	−0.36	−0.31	−**0.52**
ARAm_e	−**0.93**	−0.17	**0.51**	0.19	**0.89**	**0.81**	1.00	**0.97**	0.27	−0.10	−**0.48**	−**0.45**	−**0.57**
ARAf_e	−**0.91**	−0.16	**0.51**	0.20	**0.90**	**0.84**	**0.97**	1.00	0.25	−0.18	−**0.49**	−**0.44**	−**0.54**
AC1_e	−0.32	−0.31	−0.19	−0.29	0.32	0.33	0.27	0.25	1.00	**0.57**	−0.29	−**0.46**	−**0.40**
AC2_e	0.03	0.26	−0.06	−0.04	−0.11	−0.12	−0.10	−0.18	**0.57**	1.00	0.27	0.11	0.15
ARP_d	**0.42**	**0.81**	0.31	**0.45**	−**0.49**	−0.36	−**0.48**	−**0.49**	−0.29	0.27	1.00	**0.89**	**0.80**
RMI_d	**0.40**	**0.84**	**0.45**	**0.49**	−**0.45**	−0.31	−**0.45**	−**0.44**	−**0.46**	0.11	**0.89**	1.00	**0.83**
ARR_d	**0.51**	**0.70**	0.25	**0.61**	−**0.54**	−**0.52**	−**0.57**	−**0.54**	−**0.40**	0.15	**0.80**	**0.83**	1.00

Correlation coefficients statistically significant at the significance level 0.05 are bolded

The results of abovementioned analysis are interesting. First, the changes in the efficiency of GDP-distribution may in fact influence the changes in other dimensions (excluding administrative costs), however the efficiency of poverty reduction remains resistant to it. The ARP_e dynamics are correlated only with the dynamics of other adequacy efficiency indicators or with dynamic efficiency indicators. A very similar situation characterizes the changes in ARR_e, while RMI65+_e dynamics is associated with the changes in the labour market efficiency positively: the higher the increase in labour market efficiency, the higher the increase in the efficiency of consumption smoothing measured by RMI65+. The changes in GDP-distribution efficiency are very highly correlated with the changes in the labour market efficiency. Since the GDP-D_e indicator is a destimulating factor of efficiency, the correlation is negative and it means that the greater the increase in labour market efficiency, the greater the increase in the efficiency of GDP-distribution. Together with the analogical results from Table 1, this confirms that the relation between a pension system (GDP-distribution) and the labour market is crucial for keeping pensions stable and adequate.

## Conclusions

The efficiency of a pension system is crucial, no less important than adequacy. Nowadays, when populations have been ageing, efficiency gains on importance. In the long run, efficiency determines adequacy—an inefficient system cannot be adequate. That is the main reason why I search for the method enabling the evaluation of pension system efficiency in cross-country studies. The approach proposed in this paper has some strengths, as well as some weaknesses. To the first, belong its following features: it is of a multidimensional nature, it provides for a static as well as dynamic perspective, enables comparisons of many different empirical pension systems. Since the calculation of the indicators does not require prior standardization of data, the method is more resistant to the relativeness of the measurement and comparisons in cross-section studies. This results from the fact that the method is based on the raw, not transformed, data. Therefore, it ensures that the decrease in an indicator always means the decrease in the real value of the phenomenon it measures, and vice versa, the increase in an indicator always means the increase in the real value of the variable. In the case of standardized data, their value may increase even though the raw value of the variable decreases, but less than the values of the same variables representing other objects—pension systems of other countries. However, on the other hand, the relativeness enables the analysis of the efficiency since I do not determine the border between efficiency and inefficiency of a pension system—because of simple reason—I do not know in fact where this borderline is. Therefore, the proposed approach works effectively only when at least a few pension systems are compared, or a given pension system is analyzed in a period of time, e.g. year after year. Then inference about which pension system is better and which one is worse, or when a given system is better or worse, is possible.

The relativeness in sense that I do not indicate the borderline between efficient and inefficient pension system, is one of the weaknesses of the proposed approach to the measurement. The next one results from the fact that many pension systems are still being reformed. Therefore, sometimes we actually do not know what type of pension system is analyzed. When assessing a pension system, two sides may be under consideration: the first one refers to the generation of contributors, the other one to the generation of pensioners. All are participants of this system, but, in fact, they may function in quite different systems, because of the pension reform. The another limitation of my method results from the fact that some of the applied indicators refer only to the public pension system (e.g. pension expenditure, aggregated replacement ratio, administrative cost rates), while the other ones concern the widely understood pension system, containing also benefits from individual pension schemes or other incomes paid to pensioners (e.g. relative median income ratio, at-risk-of-poverty for pensioners). Also, labour market indicators should be treated as concerning the whole pension system since not only public, but also private schemes, may affect the decision on the exit from the labour force. For this reason, the analysis of the efficiency conducted with the use of the proposed approach, may require further and deeper study on given pension systems. However, this approach supports the choice of these systems from the set containing many pension systems.

Although unfree of disadvantages, the method seems to be working. The empirical verification delivers many arguments supporting this method, as well as other conclusions referring also to the multidimensional efficiency of studied pension systems. As the most important of them, I would indicate the following. The main goal of pension systems seems to become poverty alleviation. The efficiency of ensuring protection against poverty, as well as the efficiency of reducing poverty, is very resistant to the efficiency of GDP-distribution. The opposite situation characterizes the efficiency of consumption smoothing—this is generally sensitive to the efficiency of GDP-distribution, and its dynamic is sensitive to the dynamic of GDP-distribution efficiency. As another interesting result of the study, I treat the lack of correlation between efficiency referring to the adequacy and efficiency of changes in adequacy. This not only supports the approach proposed, according to which a static evaluation should be completed by a dynamic one, but also means that there is no relationship between the level of adequacy efficiency and its dynamics. Thus, the level of present adequacy efficiency does not determine the rate at which a given country improves, or worsens this efficiency.

The efficiency evaluation with the use of many indicators, reflecting different dimensions, together with indicators characterizing the adequacy and redistribution in pension systems, sets further possible directions of research. One of them is studying the relationship between the efficiency and adequacy of pension system as well as verifying the hypothesis that “there is no necessary trade-off between economic efficiency and achievements of welfare goals” (see Headey et al. 2000), however with respect to a pension system. Further research may also concern the qualitative analysis of construction of pension systems selected according to quantitative criteria (e.g. with the use of proposed method). It could support the search for better solutions in building pension systems in the future. The analysis suggests that the Norwegian and the Icelandic pension systems seem to be the most intriguing, in terms of efficiency. The prospective research, on the basis of proposed method, may also aim at identifying the relationships between adequacy, efficiency, and redistribution in pension systems.

## Footnotes

1 Possibly replaced by employment rate of people aged 65 and over if statistical data are available.

2 The Open Method of Coordination (OMC) was created as part of employment policy and the Luxembourg process. It has been defined as an instrument of the Lisbon strategy and provides a new framework for cooperation between the Member States, whose national policies can thus be directed towards certain common objectives. Under this intergovernmental method, the Member States are evaluated by one another (http://europa.eu/legislation_summaries/glossary/open_method_coordination_en.htm).

3 The single linkage method and complete linkage method is used to check whether there are significant differences in the dendrograms. The results are very similar.

4 Data on this indicator are available only for males and females separately. Therefore, in the case of this variable in fact two variables are used: ARAm_e for males and ARAf_e for females.
